# Clinical significance of personalized excision of AALTF in progressive collapse foot deformity: a retrospective cohort study

**DOI:** 10.3389/fsurg.2025.1669680

**Published:** 2025-11-28

**Authors:** Shangyi Liu, Xun Shu, Bin Wang, Huarui Yang, Yi Yang, Tongzhu Bao, Tao Ye, Kangquan Shou

**Affiliations:** 1 Department of Orthopedics, The First College of Clinical Medical Science, China Three Gorges University & Yichang Central People's Hospital, Yichang, China; 2 Department of Orthopedics, The Second People's Hospital, China Three Gorges University, Yichang, China

**Keywords:** subtalar impingement, accessory anterolateral talar facet, accessory talar facet impingement, symptomatic flatfoot, progressive collapse foot deformity

## Abstract

**Background:**

It has been suggested that an accessory anterolateral talar facet (AALTF) is likely to produce accessory talar facet impingement (ATFI), resulting in sinus tarsi pain in patients with progressive collapse foot deformity. However, the appropriate strategy for AALTF is not well documented. The aim of this study is to evaluate the relationship between the ATFI and AALTF in patients with progressive collapse foot deformity and elucidate the optimal treatment approach for AALTF.

**Methods:**

Seventy patients with progressive collapse foot deformity who underwent surgery between March 2014 and October 2024 were included and split into two groups: the AALTF resection group (AALTF resection alongside concomitant flatfoot procedures) and the traditional group (only flatfoot procedures were performed). All patients underwent radiographic evaluation and completed Foot Functional Index (FFI), American Orthopedic Foot and Ankle Society (AOFAS), and Foot and Ankle Ability Measure-Sports Subscale (FAAM-SS) assessments preoperatively and again at follow-up within 24 months.

**Results:**

The AALTF resection group showed significant improvements in clinical outcome measures compared to the traditional treatment group. The AALTF resection group demonstrated higher FFI scores (preoperative mean: 41.2 ± 11.4, postoperative mean: 9.3 ± 10.6) compared to the traditional group (preoperative mean: 42.1 ± 10.7, postoperative mean: 16.6 ± 4.5; *p* < 0.001). The AALTF resection group also demonstrated higher AOFAS scores (preoperative mean: 67.3 ± 14.7, postoperative mean: 92.7 ± 8.6) compared to the traditional group (preoperative mean: 68.2 ± 13.8, postoperative mean: 81.5 ± 9.1; *p* < 0.001). FAAM-SS scores were higher in the AALTF resection group (preoperative mean: 39.6 ± 11.6, postoperative mean: 90.6 ± 11.3) than in the traditional group (preoperative mean: 44.7 ± 12.3, postoperative mean: 80.9 ± 5.1; *p* < 0.001). Ankle joint ROM improved more markedly in the AALTF resection group at final follow-up (preoperative mean: 7.3° ± 2.1° to postoperative mean: 19.6° ± 3.8°) than in the traditional group (preoperative mean: 8.7° ± 1.6° to postoperative mean: 14.1° ± 2.2°; *p* < 0.001). There were no significant differences in radiological outcomes between the two groups.

**Conclusions:**

Addressing AALTF in a symptomatic flatfoot is fundamental. A careful preoperative assessment, consisting of traumatic history investigation, deliberate physical examination, and standard radiographic evaluation (especially CT and MRI scan), can help determine the most appropriate strategy for this kind of morbidity.

## 1 Introduction

Pain in the subfibular region and/or around the sinus tarsi is normally considered a major symptom in Stage II to III flatfoot deformity (1). Sewell first described the accessory anterolateral talar facet (AALTF) as an abnormal anatomical variation of the talus (2), which has been recently recognized as a potential source of pain due to talocalcaneal impingement, especially accessory talar facet impingement (ATFI) (3).

The term accessory talar facet impingement (ATFI) is defined as the presence of focal abutting bone marrow edema (BME) of the talus and calcaneus around the AALTF on MRI, accompanied by concurrent sinus tarsi pain (4). In addition, a number of studies have demonstrated that anatomic factors, such as a smaller Gissane angle, the presence of tarsal coalition, and a larger talar inferolateral surface (TILS) angle, may be prognostic risks for sinus tarsi pain in patients with AALTF (3).

The AALTF extends contiguously from the posterior talar facet, anterior to the lateral talar process (5, 6). It is likely to produce the talocalcaneal impingement in patients with rigid flatfoot deformity. As a source of pain and a prognostic factor for postoperative failure, this anatomic variant may have clinical significance and should not be ignored by surgeons. However, the literature evaluating this feature remains scarce.

The present study aims to assess clinical outcomes in progressive collapse foot deformity (PCFD) patients with sinus tarsi pain (attributed to ATFI) who underwent one of two distinct surgical strategies: flatfoot reconstruction with or without AALTF resection. We hypothesize that patients undergoing AALTF resection will demonstrate better clinical outcomes with regard to pain relief, functional score, and eversion motion of the hindfoot, compared to those who do not undergo AALTF resection.

## 2 Methods

### 2.1 Study design and patients

**Figure 1:**
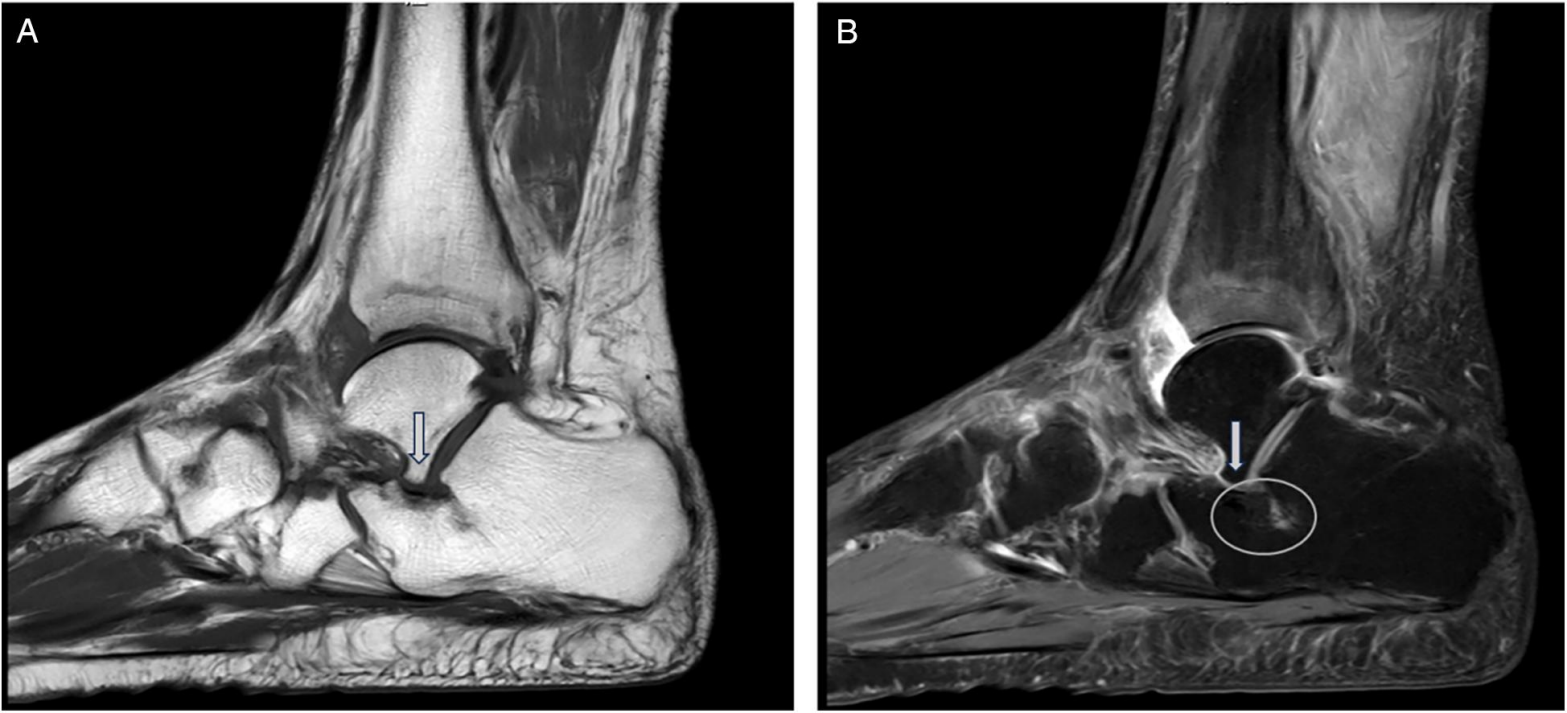
AALTF is anatomically identified as a bony protuberance arising from the lateral talar process and extending anteriorly to articulate with the calcaneus. **(A)** It is typically observed on sagittal T1-weighted MRI as a discrete osseous structure that contributes to talocalcaneal impingement in patients with flatfoot deformities (arrow). **(B)** Sagittal MRI (T2) imaging proving the presence of an osseous protuberance and the bone marrow edema located at the opposing calcaneal side of the AALTF (circle).

This retrospective cohort study was designed to analyze the outcomes of patients who underwent surgical intervention for AALTF with PCFD, with the goal of assessing the efficacy and clinical outcomes of different surgical strategies. The study was conducted between March 2014 and October 2024. A total of 70 patients (36 men and 34 women; mean age, 41.2 years; age range, 21–57 years) who had experienced persistent sinus tarsi pain for at least 3 months after failing non-operative treatment and were followed up for at least 2 years were included in the study. Sinus tarsi pain was confirmed during physical examination at the outpatient clinic by the presence of recognizable tenderness on palpation right at the sinus tarsi. Preoperative plain radiographs and magnetic resonance imaging (MRI) images were also collected for all candidates, with MRI used to confirm the presence of AALTF. AALTF is defined as a small joint that extends continuously from the posterior talar process to the lateral talar process on sagittal T1-weighted images (4) (Figure 1). All 70 patients included in the study met this radiographic criterion for AALTF.

Patients were divided into two groups based on differences in their treatment plans from previous surgical records, with grouping decisions guided by a preoperatively standardized objective assessment system: the AALTF resection group (AALTF resection along with concomitant flatfoot procedures; 19 men and 18 women) and the traditional group (only flatfoot procedures were performed; 17 men and 16 women). The standardized grouping criteria were based on three core objective indicators: (1) MRI-based assessment: AALTF size (defined as “significant AALTF” when the maximum diameter was >3 mm) and the degree of surrounding bone marrow edema (classified as mild, moderate, or severe based on T2-weighted MRI; patients with moderate-to-severe edema were included in the resection group); (2) physical examination: visual analog scale (VAS) score for sinus tarsi tenderness ≥4 points and exacerbated pain during passive hindfoot eversion; and (3) hindfoot deformity stiffness: positive Silverskiold test and subtalar joint range of motion (ROM)<10°. All preoperative assessments were independently completed by two senior orthopedic surgeons (each with ≥10 years of experience in foot and ankle surgery). In case of inconsistent opinions, a consensus was reached through departmental case discussions. The grouping method was further verified by reviewing surgical records and preoperative imaging data to ensure the objectivity and consistency of the grouping criteria. At the last follow-up, all associated data were analyzed by two additional senior surgeons who were blinded to the initial grouping criteria to avoid analytical bias. For each patient, the final radiographic result was the average of the measurements of the two surgeons. We excluded patients who responded satisfactorily to conservative treatment, as well as those with ankle osteoarthritis, severe angioneuropathy, soft-tissue infection at the surgical site, or a history of mental illness. All participants provided written informed consent, and the study was approved by the Ethics Committee of our institution.

MRI examinations were conducted using 3.0-T scanners and consisted of T1-weighted and T2-weighted images, with particular emphasis on the sagittal plane. To ensure consistency between observers, the definition of AALTF followed previously reported criteria (Accessory Talar Facet Impingement and Sinus Tarsi Pain Associated With Accessory Anterolateral Talar Facet).

### 2.2 Operative procedure and rehabilitation

**Figure 2:**
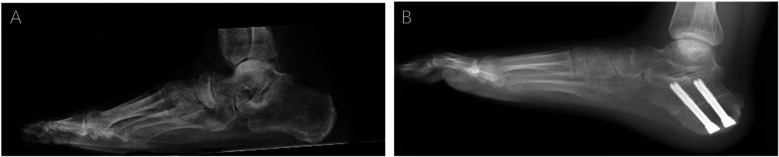
Weight-bearing lateral x-rays of the foot before and after ankle surgery. **(A)** Preoperative weight-bearing lateral x-ray of the foot: the preoperative lateral view shows significant impingement between the talus and the accessory anterolateral talar facet (AALTF), with abnormal alignment. **(B)** Postoperative image showing improved alignment and elimination of impingement after AALTF resection and flatfoot correction surgery.

With the patient in the supine position, a tourniquet was used to prevent intraoperative bleeding after the administration of general or spinal anesthesia. The following steps were performed in all cases. Group A patients underwent arthroscopic or open resection or removal of the AALTF using an oscillating saw or osteotome, accompanied by soft tissue and bony procedures for PCFD (e.g., posterior tibial tendon debridement, flexor digitorum longus transfer, medial displacement calcaneal osteotomy, and cotton osteotomy) (Figure 2). The selection of surgical approach was mainly based on AALTF-related anatomical features: an arthroscopic approach was preferred for patients with a “significant AALTF” (maximum diameter 3–8 mm) and moderate perifacet bone marrow edema (confirmed by MRI), while an open approach was adopted for those with an AALTF larger than 8 mm, severe bone marrow edema, or a concurrent calcaneonavicular coalition to ensure complete resection of the impinging structures. The AALTF was resected to a range that allowed subtalar joint fully eversion and inversion without any impingement, confirmed under direct visualization until the entire gutter of the sinus tarsi was exposed. For patients with significant preoperative limitations in ankle dorsiflexion, either Achilles tendon lengthening or gastrocnemius recession was performed based on the results of the Silverskiold test. This procedure aimed to address soft-tissue contractures and enhance postoperative range of motion, particularly in patient with severe dorsiflexion restriction. The removal of pathologically hypertrophied synovium and fibrous tissue within the sinus tarsi was also accomplished. After completing the surgical procedure, the incision was closed and ankle stability was assessed. For group B (traditional group), only the soft-tissue or bony procedures for PCFD were performed.

### 2.3 Postoperative management

The affected ankle was wrapped with an elastic bandage and elevated for 24 h. Sutures were removed 14 days postoperatively. Lower-extremity exercises, including full ankle joint range-of-motion activities, were initiated on the first day. Patients were allowed to walk partially by using crutches in early 4 weeks postoperatively. Passive varus and valgus movements of the ankle joint were prohibited for 6 weeks postoperatively. Full weight-bearing was allowed after 6 weeks postoperatively.

### 2.4 Evaluation and measurements

**Figure 3:**
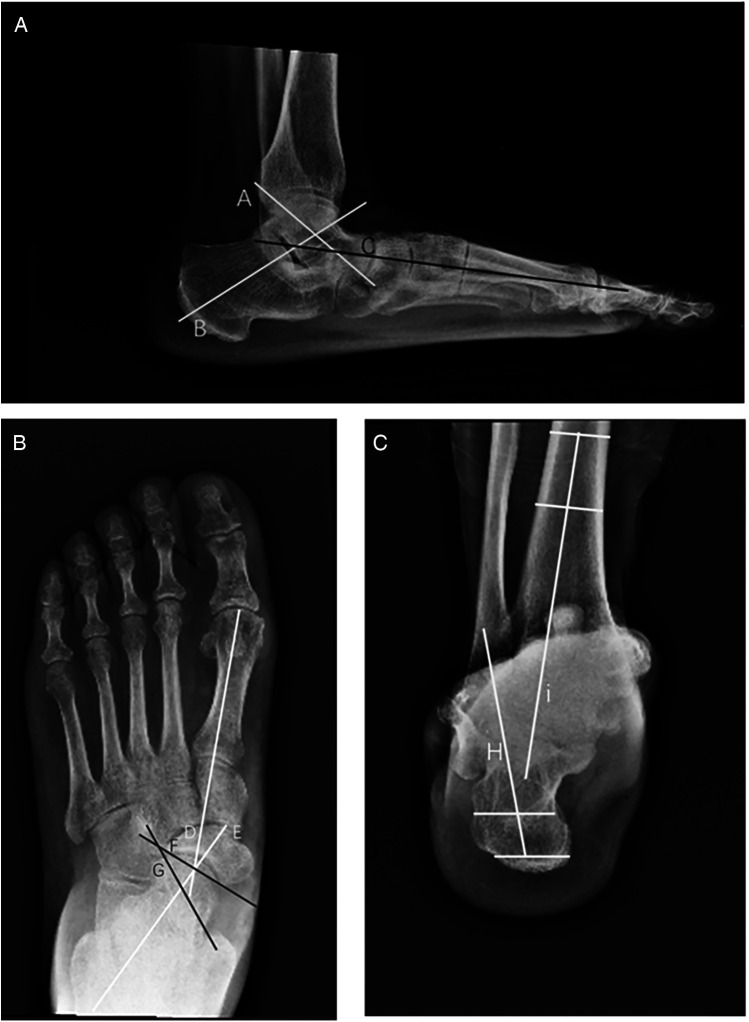
Diagram of various angles in adults with acquired flatfoot deformity. **(A)** Lateral view: anteroposterior talocalcaneal angle (kite angle)—the angle between the central axes of the calcaneus and talus (A, B); lateral talus-first metatarsal angle (Meary angle)—the angle between the central axis of the talus and the central axis of the first metatarsal (A–C). **(B)** Anteroposterior view: anteroposterior talus-first metatarsal angle (TM1)—the angle between the central axis of the first metatarsal and the central axis of the talus (D, E); talonavicular coverage angle (TNCA)—the angle formed by the central axis of the talus and the edge line of the navicular joint surface (F, G). **(C)** Hindfoot coronal alignment views: Saltzman's view hindfoot moment arm (HMA)—the distance between the two points (H, I) is HMA.

Patients were followed at 4 weeks, 3, 6, 12, and 24 months postoperatively. Pre- and postoperative AP and lateral weight-bearing foot radiographs, as well as hindfoot alignment views, were reviewed. Several parameters were measured by two independent observers, including the anteroposterior talocancaneal angle (kite angle), anteroposterior talus-first metatarsal angle (TM1), talonavicular coverage angle (TNCA), Saltzman’s view hindfoot moment arm (HMA), and the lateral talus-first metatarsal angle (Meary angle) (Figure 3) (7). For each parameter, the change was calculated as the difference between the pre- and postoperative values. The two investigators received training on unified measurement standards. At the final follow-up, ROM (plantar flexion and dorsiflexion) was measured by a goniometer. The Foot Functional Index (FFI) (8), the American Orthopaedic Foot and Ankle Society (AOFAS) Hindfoot Score, and the Foot and Ankle Ability Measure-Sports Subscale (FAAM-SS) were also assessed. Interevaluator consistency was verified using the intraclass correlation coefficient (ICC), with ICC values >0.85 for all indicators. For each patient, the final value was the average of the measurements taken by the two investigators to further minimize random error.

### 2.5 Statistical analysis

Statistical analysis was performed using SPSS version 19.0 (IBM, Chicago, IL, USA). Normally distributed data were expressed as mean ± SD, and comparisons between the two groups were made using the independent-samples *t*-test. *P-*values less than 0.05 were considered statistically significant.

## 3 Results

The mean follow-up duration was 28 ± 6.3 (range 21–37) months. Patient demographics and comorbidities for these two groups are outlined in Table 1. There was no statistically significant difference between the two groups.

**Table 1:** Patient demographics.

Variables	AALTF resection	Traditional	*P*
	(*n* = 37)	(*n* = 33)	
Age, year [mean (SD)]	43.5 (7.6)	46.3 (6.2)	>0.05 (0.162)
BMI, kg/m^2^ [mean (SD)]	21.3 (2.7)	20.2 (3.1)	>0.05 (0.245)
Bilateral [*n* (%)]	12 (33.3)	12 (37.5)	>0.05 (0.075)
Comorbidities [*n* (%)]			
Hypertension [*n* (%)]	7 (5.4)	4 (12.1)	NA
Diabetes [*n* (%)]	3 (8.1)	3 (9.1)	NA
Smoker [*n* (%)]	3 (8.1)	2 (6.1)	NA
Obesity (BMI > 30 kg/m^2^)	0 (NA)	0 (NA)	NA

AALTF, accessory anterolateral talar face; BMI, body mass index; NA, not applicable.

Ankle dorsiflexion improved significantly after surgery in the AALTF resection group, whereas no significant change was observed in the traditional group. Plantarflexion ROM was comparable between the two groups (*P* = 0.77), while dorsiflexion ROM was greater in the AALTF resection group (*P* = 0.032) at the final follow-up (Table 2).

**Table 2:** Ankle ROM between the AALTF resection group and the traditional group.

Outcome measure	Evaluation stage	AALTF resection	Traditional	*P*
Ankle dorsiflexion (°)	Preoperatively	7.3 ± 2.1	8.7 ± 1.6	>0.05 (0.168)
	Final follow-up	19.6 ± 3.8	14.1 ± 2.2	<0.05 (0.028)
Ankle plantarflexion (°)	Preoperatively	15.3 ± 4.4	14.3 ± 2.5	>0.05 (0.371)
	Final follow-up	26.1 ± 5.2	23.3 ± 2.7	>0.05 (0.314)

AALTF, accessory anterolateral talar face.

Unless otherwise noted, values are mean (SD).

Review of the radiographic parameters demonstrated that patients who underwent AALTF resection demonstrated similar postoperative radiographic changes with regard to anteroposterior talocancaneal angle (kite angle), anteroposterior TM1, TNCA, Saltzmans' view HMA, and lateral talus-first metatarsal angle (Meary angle) compared with the traditional group (*P* > 0.05; Table 3).

**Table 3:** Radiographic findings of the AALTF resection group and the traditional group at the final follow-up.

Radiographic parameters	AALTF resection	Traditional	*P*
Anteroposterior talocancaneal angle, kite angle (°)	27.6 ± 5.4	29.1 ± 3.8	>0.05 (0.371)
Anteroposterior talus-first metatarsal angle (TM1) (°)	7.2 ± 4.3	8.7 ± 2.9	>0.05 (0.402)
Talonavicular coverage angle (TNCA) (°)	15.6 ± 3.1	14.2 ± 5.1	>0.05 (0.197)
Lateral talus-first metatarsal angle (Meary angle) (°)	2.2 ± 0.8	2.8 ± 1.4	>0.05 (0.431)
Hindfoot moment arm (HMA) (mm)	12.3 ± 3.7	10.7 ± 2.9	>0.05 (0.185)

AALTF, accessory anterolateral talar face.

Unless otherwise noted, values are mean (SD).

In both groups, the FFI, AOFAS, and FAAM scores showed significant improvement compared to preoperative values (*p* < 0.01). At the final follow-up, the FFI and AOFAS scores were superior in the AALTF resection group (*p* < 0.05). In addition, the FAAM score was also significantly higher in the AALTF resection group (*p* < 0.05) (Table 4).

**Table 4:** Clinical outcomes of the AALTF resection group and the traditional group.

Outcome measures	AALTF resection	Traditional	*P*
Preoperatively			
FFI	41.2 ± 11.4 (21–52)	42.1 ± 10.7 (24–50)	>0.05 (0.512)
AOFAS	67.3 ± 14.7 (46–78)	68.2 ± 13.8 (39–80)	>0.05 (0.419)
FAAM-SS	39.6 ± 11.6 (21–88)	44.7 ± 12.3 (27–86)	>0.05 (0.437)
Final follow-up			
FFI	9.3 ± 10.6 (0–31)	16.6 ± 4.5 (0–42)	<0.05 (0.015)
AOFAS	92.7 ± 8.6 (75–99)	81.5 ± 9.1 (52–100)	<0.05 (0.017)
FAAM-SS	90.6 ± 11.3 (56–100)	80.9 ± 5.1 (53–97)	<0.05 (0.014)

FFI, Foot Functional Index; AOFAS, American Orthopedic Foot and Ankle Society; FAAM-SS, Foot and Ankle Ability Measure-Sports Subscale.

Unless otherwise noted, values are mean (SD).

No major complications or flatfoot deformity recurrences were observed, and no revision surgeries were required at the final follow-up. Minor complications included superficial wound infections, which occurred in two patients in the traditional group and one patient in the AALTF resection group.

All patients reported being either very satisfied or satisfied with the procedure, except for two patients in the traditional group who were dissatisfied.

## 4 Discussion

The main objective of this study is to investigate the relationship between AALTF resection and the treatment and prognosis of PCFD and to emphasize the importance of AALTF in flatfoot treatment. AALTF is increasingly recognized as a critical source of lateral hindfoot pain (4, 9). It is believed that AALTF may contribute to talar impingement and lead to accessory talar facet impingement (AFTI) (9). However, it remains unclear whether AALTF resection should be considered a key surgery in the management of progressive collapse foot deformity (PCFD). Through this retrospective cohort study of 70 surgically treated PCFD patients, we revealed the critical role of AALTF in the pathogenic mechanism and evaluated the effectiveness of AALTF resection in improving clinical outcomes. Our results showed that, compared with the conventional treatment group, the AALTF group demonstrated significant improvements in AOFAS, FFI, and FAAM scores, as well as in maximum dorsiflexion angle and ankle joint range of motion. Although there were no significant differences in imaging-related parameters between the groups pre- and postoperatively, AALTF resection significantly improved pain relief and functional recovery. Subtalar joint motion emerged as a critical factor in determining the surgical approach. In patients with rigid flatfoot, AALTF resection was prioritized to alleviate impingement and restore hindfoot motion. Conversely, for patients with more flexible deformities, the decision was tailored based on symptomatology, ensuring that the surgical strategy was customized to the specific biomechanical requirements of each patient. Our study indicates that AALTF resection may benefit PCFD patients by improving ankle joint ROM, thus achieving better functional recovery and higher patient satisfaction.

In 2008, Martus (10) first reported the presence of AALTF and suggested that ATFI caused by AALTF is one of the main causes of adult rigid flatfoot (such as peroneal spastic flatfoot). In recent years, ATFI caused by the anatomical variation of AALTF has been recognized as one of the causes of sinus tarsi pain (5, 11–13). In a study by Park involving 240 patients (3), 51.7% of individuals with sinus tarsi pain had AALTF, suggesting that this anatomical variation may have significant clinical relevance. A statistical survey of clinical MRI findings showed that among 120 patients with ATFI, AALTF was present in 62 patients, which far exceeded its association with calcaneal cysts and calcaneal cortical thickening. AALTF was closely related to localized bone marrow edema in patients with ATFI (3). Further research has shown that approximately 31.55% of adult patients with flatfoot have AALTF, and nearly 50% of those with AALTF develop ATFI (14). If the ligament thickens or becomes stiff, or if surrounding tissues become inflamed, particularly in patients with flatfoot, where flatfoot altered ankle biomechanics increase friction between AALTF and other structures, ATFI is more likely to occur. We believe that the presence of AALTF may reduce the anterolateral space of the ankle, especially during ankle motion. Our results show that removal of the AALTF not only improves ankle mobility but also changes the symptoms of patients.

Currently, there is no unified standard for treating ATFI, and most existing studies describe AALTF resection as part of the treatment strategy (4, 10, 15). Researchers such as Martus (10) and Niki (4) have reported positive outcomes following AALTF resection in both children and adults. However, the necessity of AALTF resection remains controversial, and there is limited research comparing postoperative functional outcomes between patients with or without AALTF resection. Furthermore, despite the lack of prospective comparative studies, postoperative symptom deterioration following AALTF resection has been rarely reported.

In our retrospective study, excision of the AALTF significantly improved postoperative function, as evidenced by higher postoperative functional scores and increased ankle mobility. These improvements were observed despite both groups undergoing the same basic procedure for flatfoot correction, with or without AALTF excision. Patient-reported outcomes suggested that AALTF excision provided additional benefits, particularly in terms of pain relief and overall comfort. However, despite these clinical benefits, no substantial changes were observed in postoperative radiologic parameters. This discrepancy highlights the complex relationship between functional outcomes and radiologic changes. We speculate that the improvement in clinical symptoms after AALTF resection is primarily due to a reduction in dynamic soft-tissue impingement rather than changes in static joint anatomy. Although AALTF resection may reduce soft-tissue impingement, thereby relieving pain and improving function, it may not alter bone structure or joint alignment sufficiently to be detected on MRI.

For adolescent patients, joint-preserving surgeries have become the primary treatment method (16, 17). These surgical methods not only effectively correct deformities and achieve satisfactory therapeutic outcomes but also do not affect the growth and development of young patients' feet (18, 19). However, in our study, the decision to perform AALTF resection or subtalar joint fusion was individualized. This choice was based not only on the patient's age but also on the condition of the subtalar joint (including the presence and extent of degeneration), as well as the complexity of flatfoot correction. Specifically, in cases where the correction of hindfoot alignment was particularly challenging—such as in rigid flatfoot deformities—subtalar joint fusion was performed to achieve a stable and functional alignment, making the surgery more feasible. In contrast, for patients with less severe degeneration and a more flexible flatfoot deformity, AALTF resection was preferred to preserve joint mobility and potentially provide better long-term outcomes. Similarly, the study by Cass and Camasta (20) supports this approach, demonstrating that AALTF resection can yield better long-term outcomes in younger patients compared to fusion surgery (21). Based on this evidence and given the absence of arthritic changes in many of our patients, we prefer AALTF resection when appropriate. This personalized treatment strategy underscores the innovation in our approach to flatfoot correction, tailoring surgical decisions to the specific needs and clinical characteristics of each patient.

**Figure 4:**
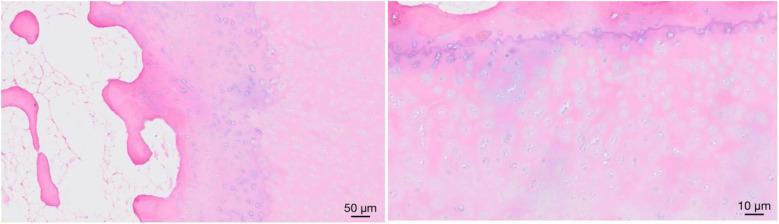
Histological examination of the surgical specimen showing the formation of fibrocartilage caps at the tissue edges, confirming the formation of AALTF.

To confirm the presence of AALTF, H&E staining was performed on the excised tissue samples during surgery. Histological analysis revealed densely packed, orderly arranged collagen fibers within the excised tissue, resembling the structure of fibrous connective tissue. In several samples, fibrocartilage caps were observed at the entheses (the attachment sites of tissue to bone) (Figure 4), suggesting the presence of AALTF as a distinct anatomical structure, although its exact classification may differ from that of typical ligament tissue.

In summary, this study focuses specifically on PCFD—a distinct clinical entity characterized by progressive biomechanical alterations—to explore the association between AALTF resection and postoperative functional outcomes. A careful preoperative assessment, including a thorough traumatic history investigation, deliberate physical examination, and standard radiographic evaluation (especially CT scan), can help determine the favorable strategy for this kind of rare case. Moreover, the surgical plan should be customized to fit the patient's needs to render the optimal outcome.

## Data Availability

The original contributions presented in the study are included in the article/Supplementary Material, further inquiries can be directed to the corresponding authors.
